# Th17 Cells Paradoxical Roles in Melanoma and Potential Application in Immunotherapy

**DOI:** 10.3389/fimmu.2019.00187

**Published:** 2019-02-08

**Authors:** Chen Chen, Feng-Hou Gao

**Affiliations:** Department of Oncology, Shanghai 9th People's Hospital, Shanghai Jiao Tong University School of Medicine, Shanghai, China

**Keywords:** Th17 cell, melanoma, tumor microenvironment, immunotherapy, adoptive cell transfer

## Abstract

The progressive infiltration of immune cells is associated with the progression of melanoma. Specifically, Th17 cells in melanoma microenvironment have both antitumor and protumor effects. It is now necessary to understand the contradictory data associated with how Th17 cells play a role in melanoma. This review will summarize the current knowledge regarding the potential mechanisms that may be involved in the effects of Th17 cells in melanoma progression. Currently, since adoptive transferring Th17 cells has been successful in eradicating melanoma in mice, it offers promise for next-generation adoptive cell transfer, as *ex vivo* expanded stemness-like memory Th17 cells which are induced by distinct cytokines or pharmacologic reagents may be infused into melanoma patients to potentiate treatment outcome.

## Introduction

Melanoma is a tumor originated from melanocytes, specialized pigmented cells that are mainly resident in the skin (1, 2). Worldwide, it is estimated that around 287,723 (1.6%) cases of all newly diagnosed cancers are cases of melanoma of the skin, and about 60,712 cancer deaths are due to melanoma of the skin, based on cancer statistic carried out in 2018 (3). Melanoma can be cured by surgical resection if it is diagnosed early, and the 5-year survival rate is over 89% (4). However, once melanoma has spread, it rapidly becomes life-threatening (5). The treatment options for patients with advanced, unresectable, or metastatic melanoma, especially BRAF-V600E/K mutant melanoma which comprises ~50% of the cases, have changed dramatically over a short period of time (6). BRAF inhibitors (Vemurafenib and Dabrafenib) and MEK inhibitors (Trametinib and Cobimetinib) have been approved by the FDA for treatment of advanced-stage melanoma patients with BRAF-V600-mutant (7, 8). In patients with BRAF-mutant, BRAF-inhibitor-refractory disease checkpoint inhibitors against PD-1 (pembrolizumab and nivolumab) and CTLA4 (ipilimumab) have demonstrated efficacy (6, 7, 9, 10). Nevertheless, 10–15% of patients treated with ipilimumab experienced a range of inflammatory side effects, also called immune-related adverse events (irAE) that even lead to death sometimes (4, 11, 12). So, development of new therapeutic approaches and optimization of current therapeutic approaches would be of great importance in the field of melanoma therapy research.

Extensive literature describes how immune cells recruited to the tumor microenvironment can exert critical functions in tumor development and progress (13). Specifically, the roles of T cells in cancer are of great interest. Apart from CD8^+^ cytotoxic T lymphocytes, CD4^+^ T cells are essential components of cell-mediate immunity. Th17 cells, as a third subset of CD4^+^ T cells, are developmentally distinct from Th1 and Th2 lineages(14, 15). IL-6, TGF-β, and IL-1β are required for Th17 cells differentiation from naïve CD4^+^ T cells and IL-21, IL-23 contribute to their maintenance (16–18). These cytokines induce signal transducer and activator of transcription 3 (STAT3) activation and expression of the major transcription factors of Th17 cells retinoic acid-related orphan receptor (ROR)γt, RORα as well as other transcriptional factors (19, 20), and then promote expression of IL-17, IL-21, IL-22 (21). It is well known that Th17 cells play an important role in inflammation disease (22, 23). And accumulating evidence suggest that Th17 cells present in tumors, including melanoma. Moreover, progressively greater number of infiltrating Th17 cells were appreciated during the development of melanoma from common melanocyte nevi to dysplastic nevi to malignant melanoma (24). Currently, many research groups have made great efforts to reveal the effects of Th17 in melanoma. Herein, we focus on the mechanisms of Th17 cells accumulation and function in melanoma and discuss ways to manipulate Th17 via cytokines and pharmaceutics to potentiate treatment outcomes in patients.

## Major Mechanisms Regulating Th17 Cell Accumulation and Expansion

### Mechanisms of Th17 Cells Recruitment in Melanoma Microenvironment

Recent studies have suggested the potential mechanisms responsible for the recruitment of Th17 cells in melanoma microenvironment. Chemokines secreted by melanoma cells and the cells within melanoma microenvironment play an important role in recruiting immune cells with corresponding receptors to melanoma microenvironment. Th17 cells express both Th1-associated (CCR2, CXCR3, CCR5, and CXCR6) and Th2-associated (CCR4) trafficking receptors. In addition, Th17 cells express non-lymphoid tissue trafficking receptors (CCR4, CCR5, CCR6, CXCR3, and CXCR6) as well as homeostatic chemokine receptors (CD62L, CCR6, CCR7, CXCR4, and CXCR5) that are involved in T cell migration to lymphoid tissues (25). However, tumor-infiltrating Th17 cells may have different chemokine receptors in different tumor contexts. In melanoma, tumor-infiltrating Th17 cells express CCR2, CCR4, CCR5, CCR6, CCR7, and CXCR3 (26).

**Figure 1:**
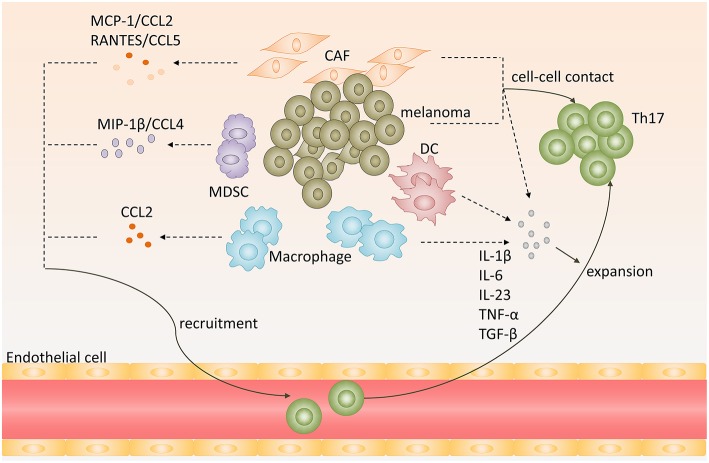
Recruitment, expansion of Th17 cells in melanoma microenvironment. Chemokines, including CCL2, CCL5, CCL4, secreted by melanoma, cancer-associated-fibroblasts (CAF), myeloid-derived suppressor cells (MDSC) or tumor-associated macrophages (TAM), promote Th17 cells recruitment to melanoma. Melanoma cells, CAFs, TAMs, and DCs produce proinflammatory cytokines, such as IL-1β, IL-6, IL-23, TNF-α, and TGF-β, and provide cell-cell contact that promote expansion of Th17 cells.

Work by Peng's group showed that tumor-derived fibroblasts secreted MCP-1 (known as CCL2), the ligand for CCR2 or CCR4, and RANTES (known as CCL5), the ligand for CCR1, CCR3, or CCR5. And they both mediated the recruitment of Th17 cells in tumor microenvironment (26). When triggering TLR and Nod signaling to increase the expression of MCP-1 and RANTES by melanoma cells and tumor-derived fibroblasts, they found that the chemotactic activity of Th17 cells was enhanced. This result suggests that chemokines, involved in TLR and NOD signaling, secreted by melanoma cells and tumor-derived fibroblasts could lead Th17 cells recruiting to melanoma sites (Figure 1) (26).

Tumor-associated macrophages (TAMs) are also involved in Th17 cells infiltration. Researchers used modified melanoma-condition (MCM) to differentiate human monocytes to macrophages and they found that these MCM-induced macrophages strikingly increased CCL2 expression (27). Furthermore, tumor-infiltrating monocytic myeloid-derived suppressor cells (MO-MDSC) and granulocytic myeloid-derived suppressor cells (PMN-MDSC) from B16-bearing mice could produce higher levels of CCL3, CCL4, CCL5 than that in control mice at melanoma sites, especially MO-MDSCs (28). And CCL4/MIP-1β, which is produced by immature myeloid cells, could recruit Th17 cells to tumor sites (Figure 1) (29).

### Th17 Cells Development, Differentiation, and Expansion in Melanoma Microenvironment

Tumor cells and tumor-derived fibroblasts promote expansion of human Th17 cells within melanoma by producing proinflammatory cytokines and providing cell-cell contact. When cocultured naïve CD4^+^ T cells isolated from human peripheral blood mononuclear cell with melanoma cells and tumor-derived fibroblasts, the percentage of Th17 cells was higher than in the medium alone. Instead, if naïve CD4^+^ T cells were separated from melanoma cells or tumor-derived fibroblasts using the transwell system, the generation of Th17 cells from naïve CD4^+^ T cells was significantly decreased compared to coculture (26). This indicated that melanoma cells and melanoma associated fibroblast could provide cell-cell contact mechanism to promote the expansion of Th17 cells, but the underlying mechanism remains to be defined.

Cytokines engaged in Th17 cells differentiation and maintenance also contribute to Th17 cells expansion in melanoma. Melanoma cells and tumor-derived fibroblasts could express high levels of IL-1β, IL-6, TGF-β, and IL-23, which provide an optimal proinflammatory cytokine milieu for Th17 cells expansion (26). Apart from melanoma cells and tumor-derived fibroblasts, dendritic cells in melanoma sites could also produce IL-6, TNF-α, IL-12p70, and IL-23 (30). In addition, macrophages are abundant leukocytes in melanoma lesions. They express high levels of IL-1β and IL-6, which may also contribute to Th17 cells expansion in melanoma (Figure 1) (31).

## Paradox of Th17 Cells Functions in Melanoma

Although Th17 cells are prevalent in melanoma microenvironment, the relationship between Th17 cells and tumor immunopathology remains controversial with both antitumor and protumor effects depicted in melanoma (32).

### Antitumor Effect of Th17 Cells in Melanoma

Th17 cells do not express neither granzyme B nor perforin and have no ability to inhibit tumor cells proliferation directly (33, 34). However, increasing evidence suggests that Th17 cells have potent antitumor effects in melanoma. Firstly, the conversion of Th17 cells toward Th1 cells may contribute to the antitumor effect of Th17 cells in melanoma. Expression IFN-γ is the main character of these Th17 cells. In metastatic melanoma patients characterized by high frequency of Th17 cells and IFNγ-secreting Th17 cells in peripheral blood before being vaccinated with therapeutic survivin-derived peptide epitopes were more likely to develop survival-specific T-cell reactivity and have higher survival rate than patients with lower frequency of these cells (35). Muranski and coworkers created a transgenic mouse expressing MHC class II-restricted T cell receptor, in which CD4^+^ T cells recognize tyrosinase-related protein 1 (TRP-1), an antigen present both in normal melanocytes and B16 melanoma cells. They found that by adoptively transferring tumor specific Th17-polarized cells into large, established B16 melanoma mice, Th17-polarized cells-mediated destruction of advanced melanoma was more effective than that of Th1 cells. And this therapeutic effect was strictly dependent on interferon-γ (IFN-γ) and IL-17 production (36). Deficient IFN-γ or IL-17A impaired Th17 cell-mediated melanoma eradication effect (37). These data suggest that Th17 cells plasticity toward Th1-like effector cells may be responsible for Th17 cells antitumor efficiency.

In addition, Martin-Orozco reported that Th17 cells within melanoma microenvironment enhance antitumor effect through recruiting other leukocytes into tumor (38). They found the expression of CCL2/CCL20 was significantly increased in lung cell fraction containing both tumor and lung cells. Further analysis revealed that CD11c^+^ DC, CD4^+^, and CD8^+^ T cells were greatly increased in Th17-treated mouse compared to control mice with metastatic melanoma in lungs (38). These results suggested that tumor-infiltrating Th17 cells stimulated tumor tissues to express CCL2/20 for recruiting various inflammatory leukocytes, such as DCs, CD4^+^, and CD8^+^ T cells to induce antitumorimmunity (34, 38).

**Figure 2:**
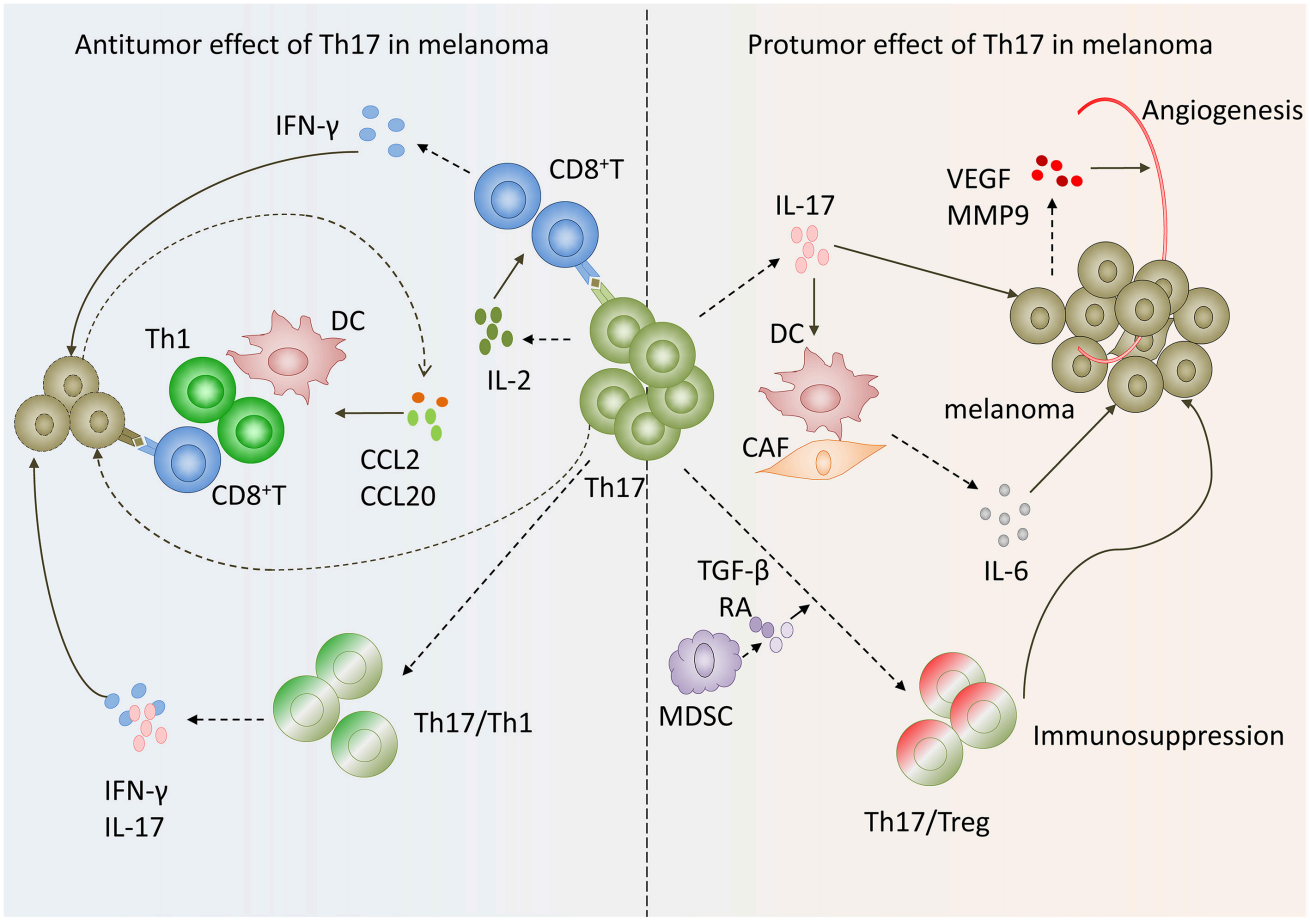
Paradox of Th17 cells functions in melanoma. On the one hand, Th17 cells in melanoma exert antitumoral function via inducing effector cells recruitment and activating tumor-specific cytotoxic CD8+T cells as well as transform to Th1 phenotype. On the other hand, Th17 cells exhibit protumor function by promoting angiogenesis, melanoma cells proliferation and phenotype change toward Tregs.

Moreover, Th17 cells can exert an antitumor effect by augmenting CD8^+^ T cells. Martin-Orozco and coworkers found that IL-17A-deficient mice were more likely to develop lung melanoma. Adoptive transferring tumor-specific Th17 cells prevents tumor development. The same group also found that therapy using Th17 cells elicited a remarkable activation of tumor-specific CD8+T cells, which were indispensable for the antitumor effect (38). In a recent study, researchers used RORγ agonist to prime TRP-1 transgenic Th17 cells and Pmel-1 TCR transgenic CD8+ T cells *ex vivo* and found these cells could effectively regress melanoma compared with those untreated Th17 cells. When co-infused with equal numbers of TRP-1, Th17 cells, and Pmel-1 Tc17 cells in mice with established melanoma, the antitumor effect was greatly enhanced. These data are consistent with previous reports, further confirming that Th17 cells can exert antitumor function by augmenting CD8^+^ T cells (39). The underlying mechanism of antitumor immunity and CTL activated by Th17 cells may be that Th17 cells stimulated CTL response via IL-2 and peptide/major histocompatibility complex (pMHC)-I, which can be recognized by CD8^+^ T cells and induce CD8^+^ T activation, based on the fact that IL2^−/−^ Th17 cells and K^b−/−^ (without MHC I) Th17 cells lost their antitumor immunity (Figure 2) (34).

### Protumor Effect of Th17 Cells in Melanoma

Despite some studies demonstrating an antitumor role of Th17 cells in melanoma, several lines of evidence suggest that Th17 cells can also have potent protumor effect in melanoma. BRAF mutation has been attributed to a reduced apoptosis, increased invasiveness and increased metastatic behavior (40). And emerging data is revealing the existence of at least two divergent immune phenotypes in melanoma. One type is the Th17 immune phenotype (Class A) with prevalent expression of cancer testis antigens, over-expression of WNT5A, enhanced cyclin activity and poor prognosis. The second class (B) Th1 immune phenotype is associated with a more differentiated status, a higher responsiveness to immune cytokines and better prognosis (41). The question whether these two different phenotypes depend upon the genetic background had been explored by Francesco M Marincola' group. When performing class comparison between BRAF mutant and wild-type metastatic melanoma samples, metastases showing a Th17 phenotype were preferentially BRAF mutated. Moreover, some genes differentially expressed between BRAF mutant and wild-type samples were related to IL-17 pathway. So Th17 cells may also have a potent protumor effect in malignant melanoma (42, 43).

Firstly, the expression of IL-17 by Th17 cells has been reported to be associated with tumor angiogenesis in melanoma. In IFN-γ deficient mice, the expression levels of vascular endothelial growth factor (VEGF) and MMP9 were up-regulated in melanoma cells. The expression of both VEGF and MMP9 were reduced in IFN-γ^−/−^IL-17^−/−^ mice (37). These data suggested that IL-17 may promote angiogenesis in melanoma. This has also been confirmed by Yan's laboratory. They found that expression levels of CD31 and MMP9 were strikingly lower in tumor tissues treated with Ad-si-IL17 than control. In addition, VEGF was down regulated when inhibiting IL-17A in tumor tissue (44). The underlying mechanism may be that IL-17 promote STAT3 activity via increasing its phosphorylation in melanoma cells and epithelial cells (45).

Secondly, Th17 cells promote tumor proliferation and survival. Lin Wang group reported that IL-17 enhanced melanoma growth due to its direct effects on IL-17 receptors expressing cells, such as melanoma cells, fibroblasts, endothelial cells, and DCs, via promoting their secretion of IL-6. And then IL-6 activated oncogenic STAT3 in melanoma cells and increased expression of prosurvival genes, such as Bcl-2, Bcl-xl. Therefore, Th17 cells can promote melanoma growth via IL-6-Stat3 pathway (45).

In addition, another mechanism involved in the Th17 cells protumor effect in melanoma may be the Th17/Tregs plasticity in melanoma microenvironment. Th17 cells can function as regulatory cells with the ability to suppress antitumor immunity. Th17 cells undergo lineage conversion into Tregs (46, 47). And this conversion results in the intermediate phenotypes that coexpress transcript factors Foxp3 and RORγt (47, 48). Tumor infiltrating Th17 cells could secrete moderate amounts of IL-10 and TGF-β1 after CD3 Ab stimulation and express Treg cell markers Foxp3, CD25, and CTLA4 (26). These results suggested that tumor-infiltrating Th17 cells may have a dual function performing both effector and regulatory roles in melanoma microenvironment. Thus, Th17 cells may contribute to immunopathogenesis of melanoma. The underlying mechanism may involve tumor-infiltrating myeloid-derived suppressor cells, which contribute to the Th17-to-Treg conversion via secretion of TGF-β and retinoic acid (Figure 2) (49).

## Basic Strategies of Adoptive Transferring Th17 Cells in Melanoma

Immunotherapy is a cornerstone in melanoma treatment. Adoptive cell transfer therapy (ACT) is a powerful way of improving patients' antitumor immunity via administration of *ex vivo* activated, expanded and selected autologous tumor-reactive T cells (50, 51). Until today, tumor-infiltrating lymphocyte (TIL) ACT was shown to elicit an objective response of 54% and complete remission of 24% in the population of melanoma patients with extremely advanced disease who have failed multiple standard therapeutic treatments (52–54). Currently, a key problem that prevents the adoption of TIL therapy is the need to infuse a vast number of cells to generate durable results in patients (53, 55, 56). To achieve consistent clinical responses, ACT requires administration of at least 40 to 60 billion tumor-reactive T cells and in some cases (57–59), up to 100 billion cells or even more (59–62), while most methods for generating vast T cells require 2 months or even longer (53). However, enhanced *in vitro* IFN-γ releasing and cytolysis CD8^+^T clones did not induce an objective clinical response upon adoptive transfer because the cytotoxic CD8^+^T cells lose their antitumor efficacy when expansively expanded *ex vivo* (52, 63, 64). This is because CD8+T cells entry into a proapoptotic and replicative senescent state and have a reduced capacity to persist *in vivo* once they reach terminal differentiation (51, 52, 65). Moreover, less differentiated CD8^+^T cells may undergo incomplete maturation (66) or even be tolerized once encountered with the tumor specific antigen (67). Therefore, investigators focus on developing potential methods to circumvent these disadvantages by using a T cell subset that is refractory to senescence (52).

Chrystal M. Paulo' group found that three-week-expanded Th17 cells experienced robust growth *in vitro* and retained long-term eliminating melanoma efficacy with the effect of memory phenotype of CD44^hi^CD62L^low^ *in vivo*, which proved to be more efficient than Th1 and 1-week-expanded Th17 cells. Furthermore, mice transferred with long-term-expanded Th17 cells were protected from melanoma rechallenge as well as lung metastasis (52). The properties that robust expansion *ex vivo* and persist long term *in vivo* of Th17 cells may partially owe to Tcf7, as it is an essential protein in the Wnt/β-catenin pathway that is critical for stem memory T cells self-renewal and the formation of memory daughter cells (37). Group found that the transcription factor Tcf7 was constantly present in nucleus of tumor-specific Th17 cells during expansion *ex vivo* (52).

Many researchers also generate Th17 cells with stemness phenotype by adding distinct cytokines or pharmacologic reagents *ex vivo* to enhance their antitumor efficacy. When cultured in the presence of IL-1β, Th17 cells express high levels of IFN-γ and exhibit enhanced antitumor effect in mice with melanoma. In addition, low doses of TGF-β could induce stemness property of IL-1β-cultured Th17 cells (68).

**Figure 3:**
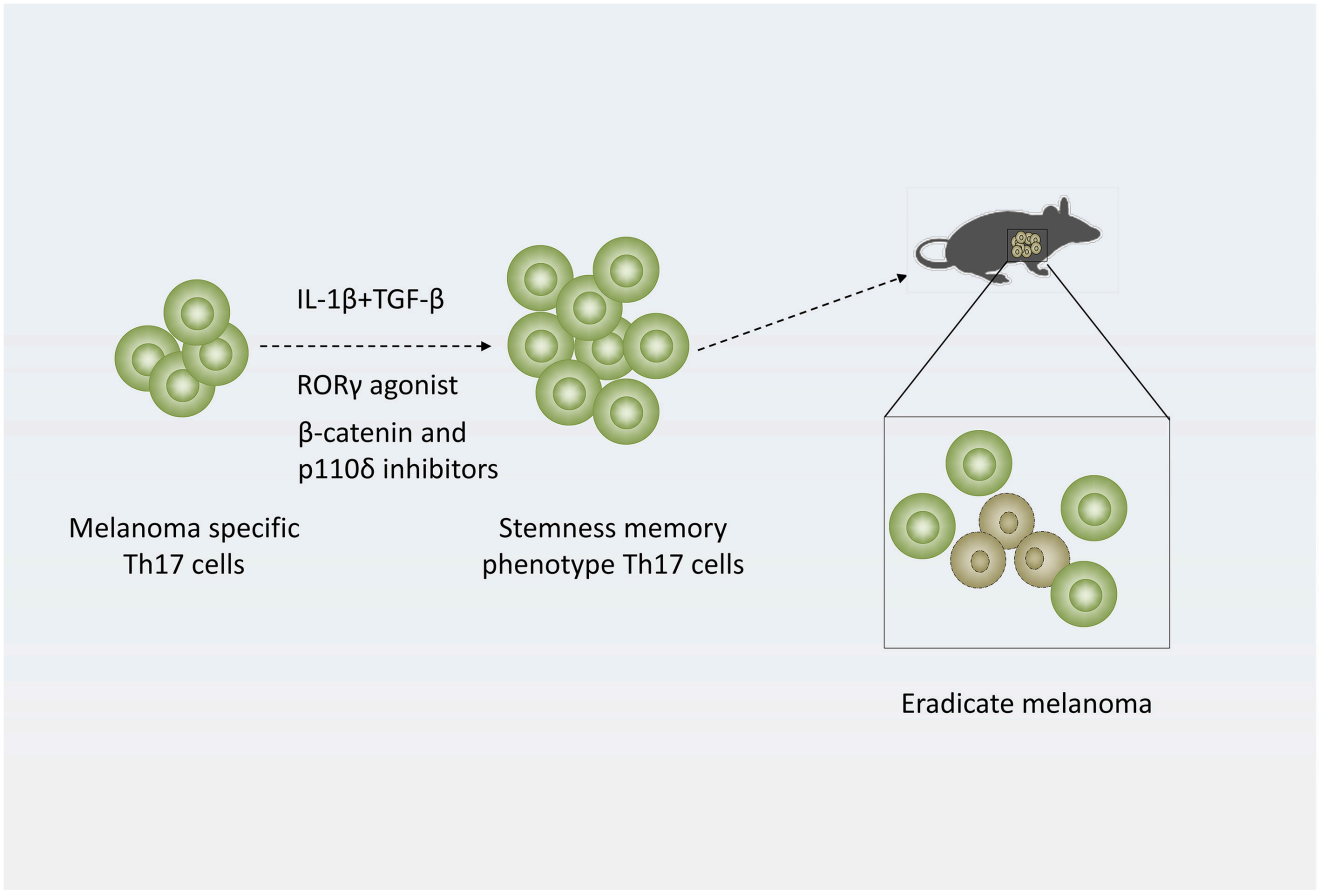
Strategy of treating melanoma with Th17 cells. Adding distinct cytokines (IL-1β and TGF-β simultaneously) or pharmacologic reagents (RORγ agonist or β-catenin and p110δ inhibitors) to Th17-polarized culture medium to generate stemness or memory Th17 cells, which are more efficient to eradicate melanoma.

In more recent studies, Chrystal M. Paulo' laboratory demonstrated that using RORγ agonist LYC-55716 *ex vivo* or β-catenin and p110δ inhibitors augments the antitumor activity of murine tumor-specific Th17 cells (39, 69). And these cells produced elevated levels of IL-17A and IFN-γ and developed a distinct memory phenotype with elevated expression of CD44 and CD62L during response to established melanoma while untreated cells mainly presented effector phenotypes in melanoma (69). Furthermore, mice previously infused with agonist-primed Th17 and Tc17 cells were protected from melanoma rechallenge (39). Their work suggests tumor specific Th17 cells treated with RORγ agonist or β-catenin and p110δ inhibitors *ex vivo* generate potent antitumor effects and persist as long-lived memory cells. This finding implicated that adoptively transferred RORγ agonist or β-catenin and p110δ inhibitors primed Th17 cells mediate long-lived memory response protecting against melanoma (Figure 3) (39, 69).

## Conclusions

Based on current studies, Th17 cells in a melanoma microenvironment have both antitumor and protumor effects. Whereas, adoptive transfer of tumor-specific Th17 cells into melanoma-bearing mice has been successful in eradicating established melanoma in mice, there still remains potential issues concerning the fact that Th17 may also contribute to tumor growth. So further studies are required to fully explore the mechanistic effect of Th17 cells in melanoma and their therapeutic value in an adjuvant therapy approach in ACT clinical trials.

## Author Contributions

F-HG and CC drafted the outline of the manuscript and conducted the literature review. CC assessed the articles and wrote the manuscript. F-HG revised the manuscript. All authors have read and approved the final version of this manuscript.

### Conflict of Interest Statement

The authors declare that the research was conducted in the absence of any commercial or financial relationships that could be construed as a potential conflict of interest.
